# MMP16 promotes tumor metastasis and indicates poor prognosis in hepatocellular carcinoma

**DOI:** 10.18632/oncotarget.20060

**Published:** 2017-08-07

**Authors:** Zhenghai Shen, Xing Wang, Xiaotian Yu, Yun Zhang, Lei Qin

**Affiliations:** ^1^ Department of General Surgery, The First Affiliated Hospital of Soochow University, Suzhou 215006, China; ^2^ Department of General Surgery, Yixing People's Hospital, Yixing, Wuxi 214200, China

**Keywords:** MMP16, hepatocellular carcinoma, prognosis, TCGA, epithelial-mesenchymal transition

## Abstract

Matrix metalloproteinase (MMPs) participates in multiple biological behaviors and plays an important role in regulating tumor invasion. However, the functions of MMP16 in hepatocellular carcinoma (HCC) remains unknown. The prognostic value of MMP16 was studied in TCGA database and validation cohort. MMP16-silencing HCC cells (HepG2 and HCCLM3) were used for evaluating cell proliferation and invasion by CCK-8 and Transwell assays. Our results showed that the MMP16 was a predictor for overall survival in patients with HCC (HR: 1.169, 95% CI: 1.034–1.321, *P* = 0.013) in TCGA database. In validation cohort, MMP16 expression was an independent predictor for survival in both univariate and multivariate analysis (*P* < 0.05). Furthermore, knockdown MMP16 weakened the cell invasive potential by inhibiting epithelial-mesenchymal transition (EMT) process. Therefore, our findings showed that MMP16 was a prognostic factor in HCC, ectopic MMP16 expression promoted invasion of HCC cells by inducing EMT process, suggesting a tumor oncogenic function in HCC and provides the potential therapeutic target for the treatment of HCC.

## INTRODUCTION

Human hepatocellular carcinoma (HCC) is the second leading cause of cancer-related death worldwide, with an estimated over half a million deaths annually [1, 2], and Chinese account for 55 % of all HCC cases [3]. The incidence has increased rapidly in Asian countries in the past years, partly due to the prevalent rate of hepatitis virus infection [4]. Surgical resection is regards as the most effective therapy for HCC. However, about 85% of patients were diagnosed with locally advanced or distant metastasis disease, and were not suitable for hepatoectomy. Moreover, clinical research has shown that > 50% of patients with HCC relapse in the follow-up time after resection [5]. Therefore, it is urgently needed for in-depth understanding of the molecular mechanisms of HCC metastasis to identify potential biomarker that may serve as new treatment targets.

The mechanisms of metastasis are multifactorial and include detachment of cancer cells from the primary tumor site, cell motility, cell to cell adherence, destruction of the basement membrane, invasion into the extracellular matrix, growth in the target tissue, and so on [6]. Destruction of the extracellular matrix is regards as a requisite initial step, because the basement membrane is though as a barrier against cancer invasion [6, 7]. Numerous studies have reported the relationship between matrix metalloproteinase (MMPs) and the invasive ability of malignant tumors [6, 8–10]. For example, MMP-2 and MMP-9 have been reported to play a key role in the invasive of various tumors by the degradation of the basement membrane [11]. MMP-9 is closely participated in capsular infiltration in HCC [6]. Recent studied demonstrated that MMP16 was highly expressed and correlated with poor prognosis in gastric cancer patients by promoting proliferation and invasion of gastric cancer cells [8]. However, the clinical significance of MMP16 in HCC has never been reported.

In presents study, we first studied MMP16 expression in The Cancer Genome Atlas (TCGA) database and validation cohort, and found MMP16 was a predictor in HCC. Functional study demonstrated that silencing MMP16 inhibited HCC cell migration *in vitro*.

## RESULTS

### Role of MMP16 in HCC patient survival in TCGA database

A total of 330 eligible HCC patients were included in the study. Table 1 summarized the clinicopathological characteristics of these patients. The median age were 61 years old (range 17 to 90). There were 226 (68.5%) patients at N0 stage, 3 (0.9%) patients at N1 stage, and 101(30.6%) patients with unclarified stage. Majority of the patients (239, 72.4%) with no distant metastases, while 3 (0.9%) patients with distant metastases, and other 88 (26.7%) patients with unknown metastases status.

**Table 1:** Clinical characteristics of patients with hepatocellular carcinoma in the TCGA

Variable		TCGA Cohort		Validation Cohort	
		*N*	%	*N*	%
Sex	Male	211	63.9	38	55.1
	Female	103	31.2	31	44.9
	Unknown	16	4.8	/	/
Age		61	17–90	59	32–76
Grade	G1	45	13.6	12	17.4
	G2	159	48.2	31	44.9
	G3	109	33.0	21	30.4
	G4	12	3.6	5	7.2
	Unknown	5	1.5	/	/
*T* stage	T1	172	52.1	46	66.7
	T2	84	25.5	14	20.3
	T3	60	18.2	8	11.6
	T4	12	3.6	1	1.4
	TX	2	0.6	/	/
*N* stage	N0	226	68.5	57	82.6
	N1	3	0.9	12	17.4
	Nx	101	30.6	/	/
*M* stage	M0	239	72.4	69	100%
	M1	3	0.9	/	/
	Mx	88	26.7	/	
AFP (ng/ml)					
	≤ 10	/	/	26	37.7
	> 10	/	/	43	62.3
Child					
	A	205	62.1	63	91.3%
	B	20	6.1	6	8.7%
	C	1	0.3	/	/
	Unknown	104	31.5	/	/
Resection type	R0	293	88.8	69	100%
	R1	15	4.5	/	/
	R2	1	0.3	/	/
	Rx	21	6.4	/	/

**Figure 1:**
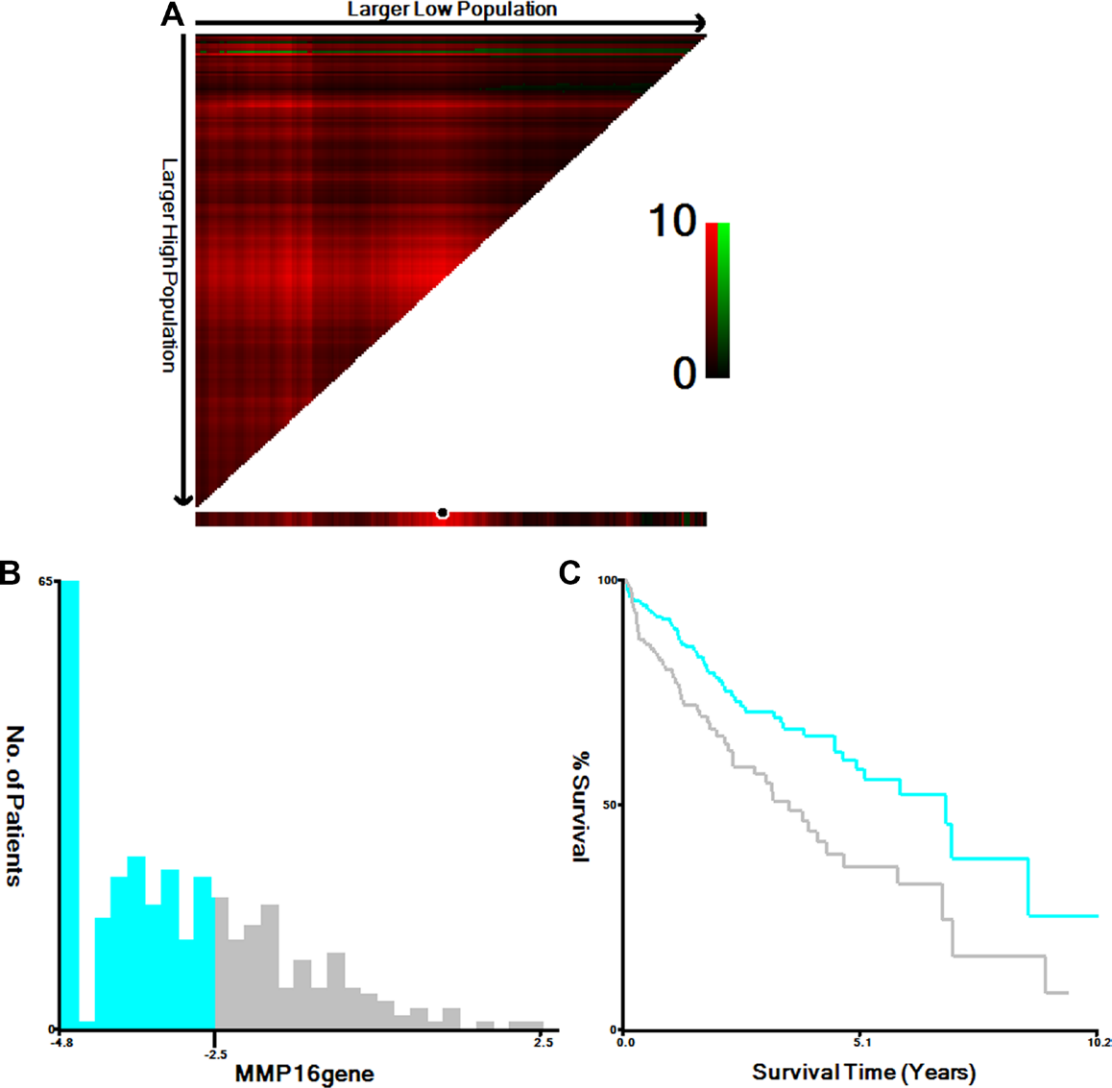
Determination of cut-off values of MMP16 expressions in TCGA database and survival analyses X-tile analysis of 5-year OS was performed using patients’ data in TCGA database to determine the optimal cut-off value for MMP16 expression. The sample of HCC patients was equally divided into training and validation sets. X-tile plots of training sets are shown in (**A**), with plots of matched validation sets shown in the smaller inset. The optimal cut-off values highlighted by the black circles in (A) are shown in histograms of the entire cohort (**B**), and Kaplan-Meier plots are displayed in (**C**). *P* values were determined by using the cut-off values defined in training sets and applying them to validation sets. (A) The optimal cut-off value for MMP16 was −2.50 (χ^2^ = 9.259, *P* = 0.002).

We first treated MMP16 as continuous variable, univariate Cox analysis indicated that MMP16 was a predictor for overall survival in patients with HCC (HR: 1.169, 95% CI: 1.034–1.321, *P* = 0.013). Then, *X*-tile program was used to determine the optimal cut-off value for MMP16 expression in term of OS to divide the patients into MMP16 high and low expression levels. There were 208 (63.0%) patients at MMP16 low expression group and 122 (37.0%) patients at MMP16 high expression levels. At the end of the last follow-up, 107 patients were died from the disease, there was significantly higher percentage of high MMP16 expression in patients died of the disease than those were live (Table 2). The 5-year overall survival were 57.8% and 36.4%, respectively, for MMP16 high and low group, which difference was also statistical significance (χ^2^ = 9.259, *P* = 0.002) (Figure 1).

**Table 2:** Relationship between survival status and the MMP16 expression

Status	MMP16 expression		χ^2^ Value	*P*
	Low (%)	High (%)		
Live	153 (73.56)	70 (57.38)	9.188	0.002
Dead	55 (26.44)	52 (42.62)		
Total	208	122		

### High MMP16 expression correlates with worse survival outcome in the validation cohort

**Figure 2:**
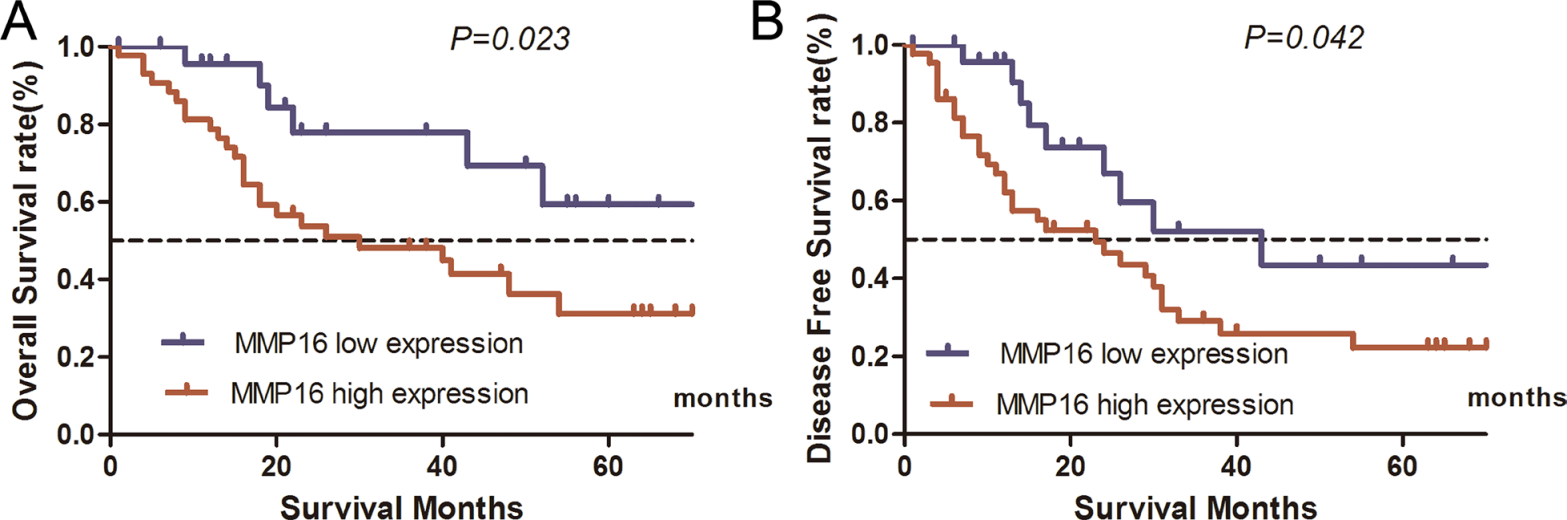
Influence of MMP16 expression patterns on overall survival and disease free survival by Kaplan-Meier analyses in the validation cohort (**A**) OS: χ^2^ = 5.172, *P* = 0.023; (**B**) χ^2^ = 4.119, *P* = 0.042.

For TCGA database included some patients received palliative resection or the quality of surgery is unknown. To get firm conclusion of MMP16 in HCC, we further evaluate the correlation between MMP16 expression and 5-year patient survival in additional 69 patients with HCC. The characteristics of these cohort were summarized in Table 1. We constructed Kaplan–Meier survival curves using overall or disease free 5-year patient survival data to analyze the cases with high and low MMP16 expression. Our analysis revealed that OS in the high MMP16 expression subgroup was 30.7% compared with 59.2% in the low expression subgroup. The log-rank analysis showed that the differences were significant (χ^2^ = 5.172, *P* = 0.023, Figure 2A). Similarly, patients with high MMP16 expression levels showed worse 5-year DFS than those with high expression (22.1% vs. 22.1%, χ^2^ = 4.119, *P* = 0.042) (Figure 2B). These data further confirmed that MMP16 may be a potential prognostic marker in clinical practice.

### Expression patterns of MMP16 in HCC

**Figure 3:**
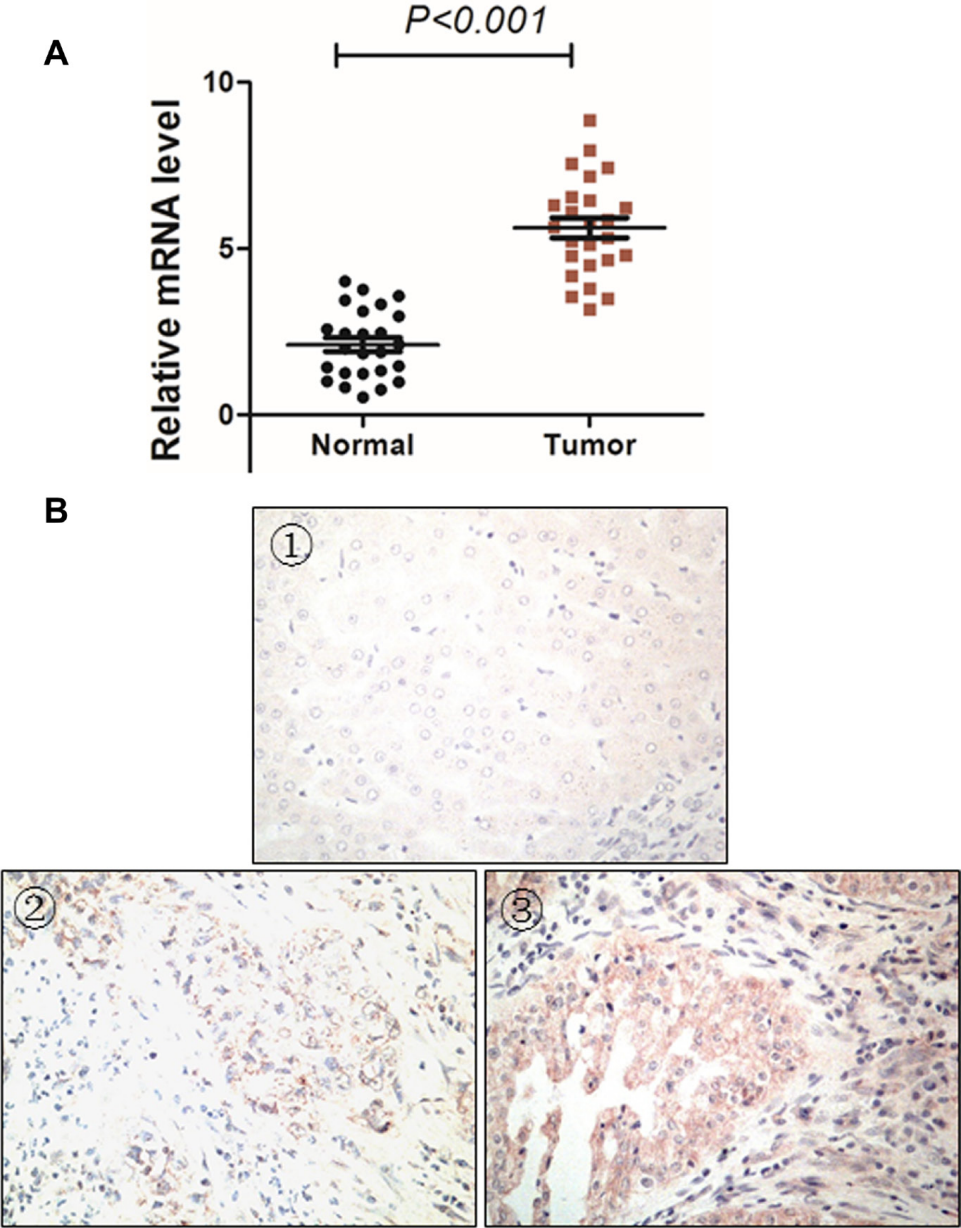
MMP16 expression patterns in HCC tissues MMP16 expression was significantly higher in tumor tissues than their adjacent normal control at both transcriptional (**A**) and protein levels (**B**). (B) displayed (①) negative MMP16 expression in normal liver tissues, (① and ②) negative (②) and positive (③) MMP16 expression in HCC tissues.

We then studied MMP16 expression in 25 paired cases cancer tissues and their adjacent normal tissues by RT-PCR and IHC study. As anticipated, the MMP16 mRNA expression levels in cancer tissues were significantly higher than their paired adjacent normal mucosa (*P* < 0.001, Figure 3A). The IHC study indicated that there was only weak MMP16 staining in normal liver tissues, while its expression was high in tumor tissues (Figure 3B). These data suggest that MMP16 may act as an oncogene in HCC. Specifically, MMP16 transcriptional expression levels were consisted with its protein expression levels.

### *In vitro* oncogenic properties of MMP16 in HCC

**Figure 4:**
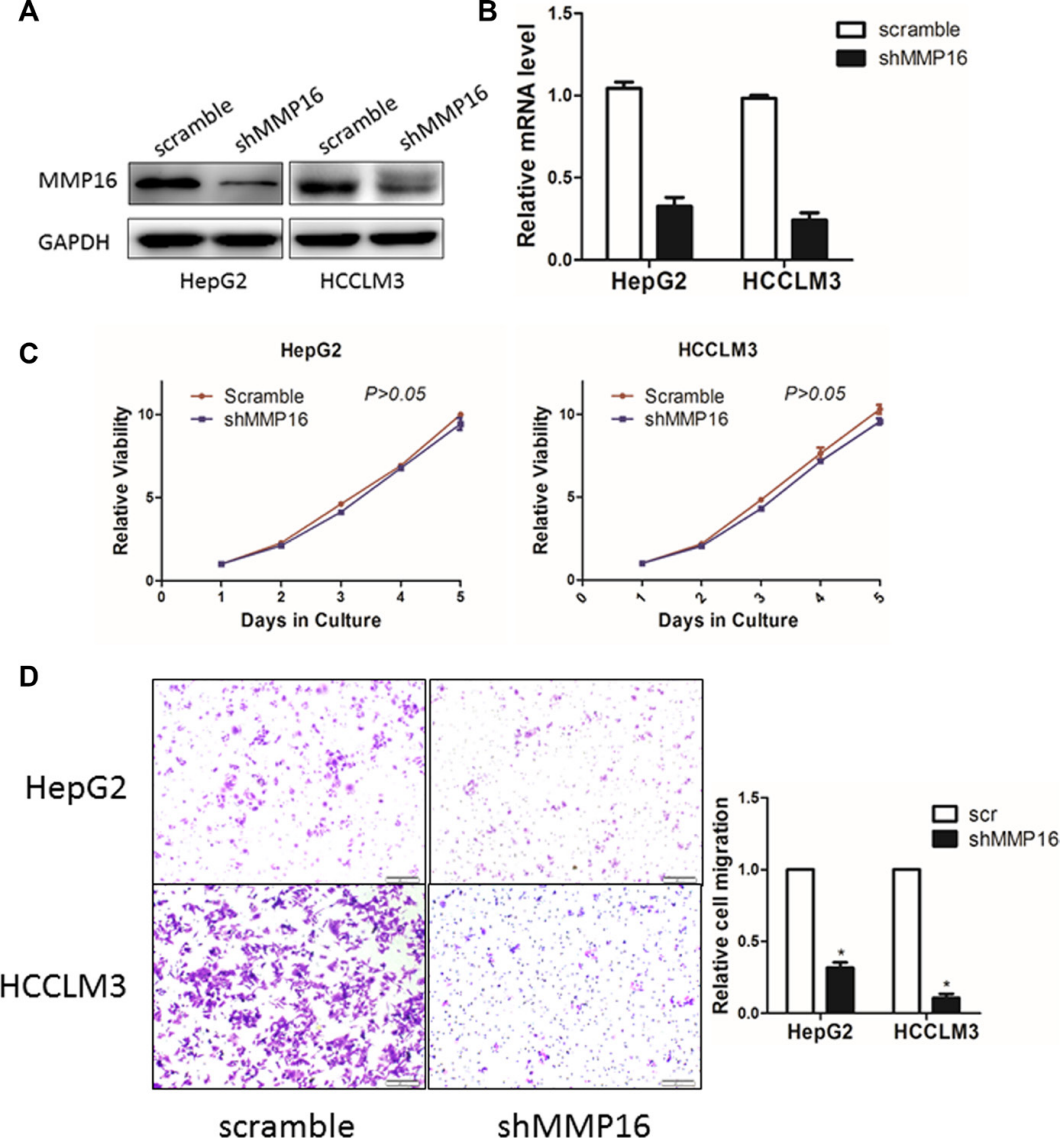
The effect of MMP16 on HCC cell proliferation and invasion The MMP16 in HepG2 and HCCLM3 cells after transfection of shRNA against MMP16 or scramble sequence was analyzed by Western blot (**A**) and RT-PCR (**B**). (**C**) Growth curves of HepG2 and HCCLM3 cells with transfected shRNAs or scramble sequence. Cell growth was determined by CCK-8. (**D**) Representative images were shown of migration of HepG2 and HCCLM3 cells via transwells measured by direct counting of trespassing cells.

We next determined whether stable knockdown of MMP16 could affect the oncogenic properties of HCC cells *in vitro*. The effect of MMP16 knockdown were determined by RT-PCR and Western blot (Figure 4A and 4B). CCK-8 assay indicated that silencing MMP16 expression did not affect the growth rate of HCC cells (Figure 4C). Then, transwell analysis was adopted to study the invasiveness in MMP16 knockdown cells. The results showed that the number of cells transmigrated across the membranes was substantially reduced in MMP16 knockdown group when compared to scramble groups (*P* < 0.05) (Figure 4D), suggesting the loss of MMP16 would impaired the migration of HCC cells.

### MMP16 induced epithelial-mesenchymal transition in HCC

**Figure 5:**
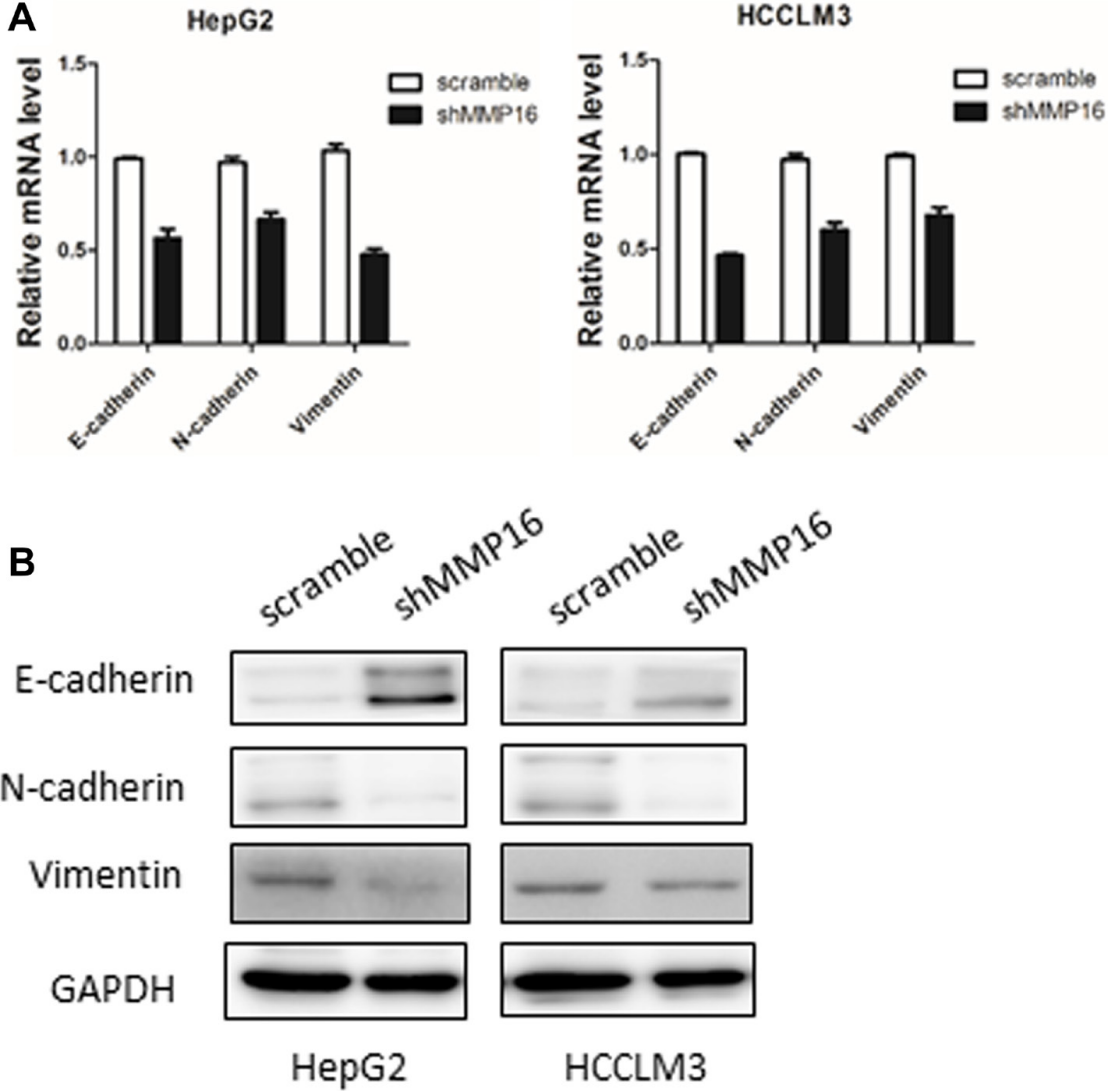
MMP16 promotes epithelial-mesenchymal transition in HCC Silencing MMP16 expression resulted in increased expression of E-cadherin and decreased expression of Vimentin and N-cadherin both on transcriptional (**A**) and protein level (**B**).

Since epithelial-mesenchymal transition (EMT) is closely related to cancer cell metastasis ability, we then examined EMT markers in MMP16 knockdown cells and their control cell lines. We found that silencing MMP16 expression lead to increase expression of epithelial cell-specific protein, E-cadherin, and repression of mesenchymal markers, Vimentin and N-cadherin at both mRNA and protein levels (Figure 5A and 5B).

## DISCUSSION

Invasion and metastasis are characteristic features of HCC and major poor prognostic factor in HCC patients [12]. Degradation of the extracellular matrix and the basement membrane is critical for invasion and metastasis. Accumulating evidence shows that MMP families involve in cancer development and progression. Furthermore, ectopic MMPs expression is associated with metastasis, invasion, and patient survival outcome. However, the function of MMP families are still not fully understood, which deserve further investigation. In order to study the function of MMP16 in HCC, we first study MMP16 in TCGA database, and found it as a prognostic factor in HCC, then we validated the prognostic value of MMP16 in additional 69 cases of HCC in our institute. Furthermore, in 25 paired HCC tumor tissues and their adjacent normal control, we found MMP16 was significantly upregulated in HCC tissues than their normal control. Also, the MMP16 transcription expression levels were consistence with their protein levels. In order to further explore the function of MMP16 in HCC, we silenced the expression of MMP16 in HCC cell lines using shRNA. An important observation of this study is that MMP16 inhibited the migration, but not proliferation *in vitro*.

Since accumulating evidences suggested that EMT is a fundamental event in in cancer progression, through which tissue epithelial cancers invade and metastasis [13, 14]. We tested whether the MMP16 promoted cancer cell invasion and metastases by way of induction of the EMT process. As anticipated, silencing MMP16 expression led to the increased protein levels of epithelial marker (E-cadherin) and decreased expression of mesenchymal marker (N-cadherin, vimentin) at both mRNA and protein levels. This observations indicated that MMP16 promotes HCC progression at least in part through inducing the EMT process.

It has been reported that ectopic MMP16 expression can promote migration and invasion of gastric cancer cells and thus cause worse survival outcome in gastric cancer [8]. MMP16 is a downstream of β-catenin gene in human gastric cancer, induction of the MMP16 expression is critical in the Wnt-mediated progression and metastasis in gastric cancer cells [15, 16], β-catenin itself is also a key regulator in EMT procedure. In melanoma, MMP16 can promote the invasion and metastasis of tumor cells by decreasing cell adhesion, inhibiting collagen alignment and inducing lymphatic invasion [16]. Therefore, it is not surprising that high MMP16 expression promoted the migration and metastasis abilities and led to poor survival outcomes in HCC.

In summary, our study reveals that MMP16 is a prognostic biomarker in HCC, MMP16 promotes the migration and metastases of HCC cells via inducing EMT process. Inhibition of MMP16 expression in HCC represents an attractive target in liver cancer therapy.

## MATERIALS AND METHODS

### Patient samples

Two cohorts of patients were included. First, we retrieved MMP16 mRNA expression levels from TCGA RNA-sequence database (https://genome-cancer.ucsc.edu/). All patients included in the study should be pathological diagnosed with HCC, have no pretreatment, and with intact OS information. Second, 69 cases of primary HCC cancer and 25 pairs of primary HCC cancer tissues and matched adjacent normal tissues who treated at the Department of Hepatology in Yixing Hospital were collected for RT-PCR and IHC study. All of the patients provided signed informed consent. The study was approved by the Ethical Committee of Yixing Hospital. We confirmed that all methods were performed in accordance with the relevant guidelines and regulations.

### HCC cell lines and cell culture

The human HCC cell lines HepG2 and HCCLM3 was originally obtained from the Chinese Center for Type Cultures Collections (CCTCC, Wuhan, China). The cell lines were cultured in RPMI 1640 media (Life Technologies, Beijing, China) and supplemented with 10% fetal bovine serum (FBS) (Life Technologies, Beijing, China). All the Cells were maintained at 37°C in a humidified atmosphere with 5% CO2.

### Stable transfection of HCC cells

Biologically active short hairpin RNAs (shRNA) were generated using the lentiviral expression vector pLKO.1-puro. The shRNA target sequence for human MMP16 was 5′-CGTGATGTGGATATAACCATT −3′. PLKO.1-scramble shRNA with limited homology with any known sequences in the human was used as a negative control. HepG2 and HCCLM3 were transfected with the pLKO.1-shMMP16 expression vector or pLKO.1-scramble.The cells stably transfected were isolated using puromycin selection to obtain stable MMP16 knockdown cells.

### Cell proliferation assay

Cell proliferation was detected using a Cell Counting Kit-8 assay (Dojindo, Kumamoto, Japan). Cells were plated in 96-well plates at 2 × 10^3^ cells/well. After 24 h culture, the HCC cells were replaced with 10% CCK-8 diluted with normal culture medium for color conversion for another 2 h. Proliferation rate was measured at 24 h, 48 h, 72 h and 96 h after seeded.

### Cell migration assay

To evaluate the migration capacity of cells, 24-well plates equipped with cell culture inserts containing 8.0 μm pore size membrane (Costar Corp., Cambridge, MA, USA) were used. Briefly, Diluted extracellular matrix gel (BD Biosciences, Bedford, MA, USA) coated the inserts preincubated for 30 min. 1 × 10^5^ cells were suspended in 100 μl of serum-free medium and placed in the upper compartment chambers. The lower chamber was filled with 10% FBS as the chemoattractant. At the end of the experiments, cells on the upper surface of the filters were wiped, and migrated cells on the lower surface were fixed in 4% paraformaldehyde and stained by 0.1% crystal violet.

### Immunoblotting, immunohistochemistry (IHC) study

IHC studies were carried out as described previously [8]. In brief, paraffin-embedded sections were subjected to routine toasting, dehydration, endogenous peroxidase inactivation and antigen retrieval. Samples were incubated with primary antibodies overnight at 4°C and secondary antibody for 1 h at room temperature. Diaminobenzidine (DAB) substrate was used for sample staining. Anti-MMP16 antibody (Abgent) was used in a dilution factor of 1:100.

### RT-PCR

The total RNA was extracted from cells using TRIzol (Invitrogen), and cDNA was synthesized using PrimeScript^®^ RT regent kit (Takara Biotechnology, Dalian, China). Real-time PCR was performed with SYBR^®^ Premix Ex Taq (Takara Biotechnology, Dalian, China). The relative gene expression levels were calculated using the RQ value. The sequences for sense (S) and antisense (AS) primers as follows: human-MMP16-S, 5′ - AGCACTGGAAGACGGTTGG - 3′, human-MMP16-AS, 5′- CTCCGTTCCGCAGACTGTA-3′, human-GAPDH-S, 5′- GCAAATTCCATGGCACCGT-3′, human-GAPDG-AS, 5′- TCGCCCCACTTGATTTTGG −3′. The relative quantitation (RQ) of the gene expression was applied to analyze the relative changes between them.

### Western blot assay

For total protein extraction, cells were washed once with phosphate-buffered saline (PBS) and lysed with RIPA buffer (RIPA Lysis Buffer, Thermo Scientific Pierce) supplemented with complete protease inhibitor tablets for 30 minutes on ice. Protein concentration was quantifed with Coomassie Plus (Bradford) Protein Assay Reagent according to manufacturer's instructions. Extracts (40 μg) were resolved on 10% SDS-PAGE and transferred to polyvinylidene difluoride membranes (Bio-rad). Membranes were probed with primary antibodies against anti-MMP16 (1:500, Abgent) and anti-GAPDH (1:5000, Santa Cruz), respectively, followed by incubation for 1 hour at room temperature with HRP-conjugated anti-rabbit Ig. All experiments were performed at least three independent times.

### Statistical analysis

X-tile 3.6.1 software [17] (Yale University, New Haven, CT, USA) was used to determine the optimal cut-off value for MMP16 expression in TCGA cohort. Survival analysis was performed using the Kaplan-Meier method and Cox regression model. Functional studies was performed in triplicate and repeated at least three times. Data were presented as mean ± standard deviation (SD). The statistical analyses were conducted with SPSS 20.0. *P*-values < 0.05 were considered statistically significant.
